# Supplementary material for: Bloodstream Infections in Critical Care Units in England, April 2017 to March 2023: Results from the First Six Years of a National Surveillance Programme
Source: Microorganisms. 2025 Jan 16;13(1):183. doi: 10.3390/microorganisms13010183 (PMC11767419; doi:10.3390/microorganisms13010183)
Supplement: Supplementary file 1 [file microorganisms-13-00183-s001.zip › microorganisms-3398689-supplementary.pdf]

Supplementary material

to ‘Bloodstream infections in critical care units in England, April 2017 to March 2023: results from the first six years of a national surveillance programme’

Table S1: Antimicrobial resistance in Gram-negative PBC, participating adult CCUs in England, April 2017 to March 2023

Antibiotic	*E. coli*		*K. pneumoniae*		*Enterobacter cloacae*		*P. aeruginosa*	
	T	%R	T	%R	T	%R	T	%R
Amoxicillin/clavulanate	1,437	51	877	31	-	-	-	-
Ceftazidime	1,208	14	750	16	261	36	516	12
Cefotaxime	785	14	491	15	180	43	-	-
3rd gen. cephalosporins*	1,208	14	750	16	261	38	-	-
Piperacillin/tazobactam	1,466	13	884	22	321	29	499	13
Ciprofloxacin	1,508	22	906	16	327	4	529	11
Gentamicin	1,523	13	910	8	328	5	470	7
Amikacin	1,107	2	718	2	261	0	424	4
Colistin	115	3	60	8	-	-	83	8
Meropenem	1,502	0	906	2	329	1	524	15

T = total number of isolates with available susceptibility results. %R = percentage of resistant isolates.
* ‘3rd generation cephalosporins’ indicates resistance to either ceftazidime or cefotaxime.

Table S2: Antimicrobial resistance in Gram-positive PBC, participating adult CCUs in England, April 2017 to March 2023

Antibiotic	*Enterococcus faecium*		Coagulase-negative staphylococci		*Staphylococcus aureus*	
	T	%R	T	%R	T	%R
Ciprofloxacin	-	-	5,357	63	1,093	11
Meticillin*	-	-	6,106	75	1,174	8
Mupirocin	-	-	3,378	43	1,107	1
Fusidic acid	-	-	5,781	62	1,141	11
Rifampicin	-	-	5,659	12	1,126	1
Gentamicin	-	-	5,697	62	1,150	4
Vancomycin	951	22	3,275	1	876	0
Teicoplanin	884	24	2,286	17	768	0
Linezolid	934	1	5,296	1	1,060	0

T = total number of isolates with available susceptibility results. %R = percentage of resistant isolates.
* ‘Meticillin’ indicates resistance to any of the following: meticillin, oxacillin, cloxacillin or cefoxitin.

Figure S1: Antimicrobial resistance in Gram-positive PBC, participating adult CCUs in England, April 2017 to March 2023, by financial year

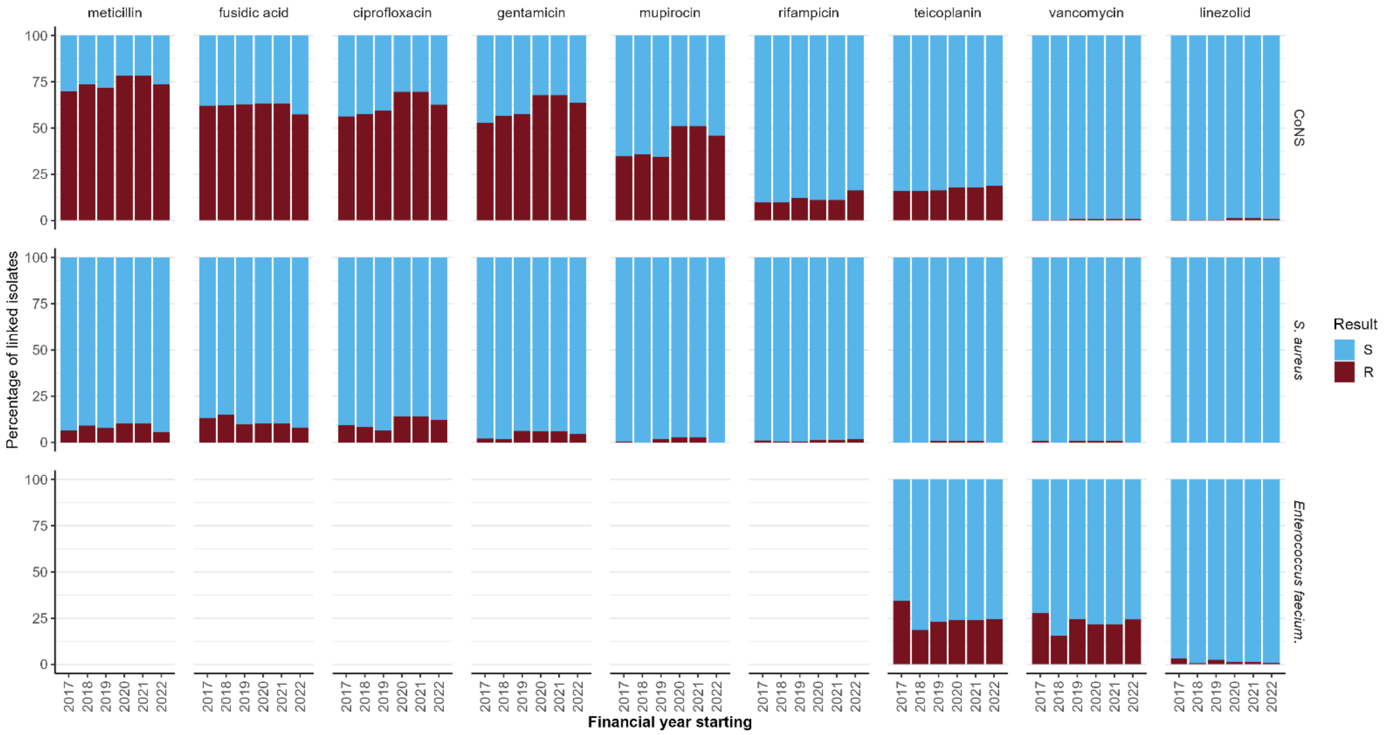

‘S’ includes ‘susceptible’, ‘susceptible, normal exposure’, ‘intermediate’ and ‘susceptible, increased exposure’). ‘R’ represents ‘resistant’. CoNS = coagulase-negative staphylococci.
